# Beans Improve Satiety to an Effect that Is Not Significantly Different from Beef in Older Adults: A Randomized, Crossover Trial

**DOI:** 10.1016/j.tjnut.2025.02.008

**Published:** 2025-02-14

**Authors:** Megan J Fluit, Brooke F Adams, Zachary J Ribau, Alison M Duncan

**Affiliations:** Department of Human Health and Nutritional Sciences, University of Guelph, Guelph, Ontario, Canada

**Keywords:** appetite sensations, beans, beef, satiety, older adults

## Abstract

**Background:**

Beans are a candidate food for increasing satiety due to their protein and dietary fiber content. Beef is a common animal protein that can increase satiety due to its protein content, which is higher than beans but does not contain dietary fiber. Dietary guidance encourages higher intake of plant-based protein foods and warrants satiety studies that compare plant and animal protein foods, which could particularly benefit the rapidly growing population segment of older adults.

**Objectives:**

To compare the effects of 2 bean varieties and beef consumed within a breakfast tortilla on satiety, food intake, and 24-h energy intake in older adults.

**Methods:**

Older adults [*n* = 35, age 72.4 ± 6.66 y, BMI (in kg/m^2^) 25.1 ± 3.25] consumed 3 breakfast tortilla test meals containing 1 serving of black beans (135 g), red kidney beans (135 g), or beef (80 g) in a randomized, crossover design. Participants rated their appetite sensations on periodic visual analogue scales, food intake was measured at an ad libitum pizza lunch meal, and 24-h energy intake was measured using weighed food records. Appetite sensation area under the curves (AUCs) were compared between treatments using repeated-measures analysis of covariance, and food intake and 24-h energy intake were compared using repeated-measures analysis of variance.

**Results:**

Fullness and satisfaction were significantly increased, while hunger, desire to eat, and prospective food consumption were significantly decreased, following consumption of the black bean, red kidney bean, and beef test meals. Appetite sensation AUCs, ad libitum pizza intake, and 24-h energy intake did not significantly differ between the test meals.

**Conclusions:**

These results demonstrate that beans improve satiety to an extent that is not significantly different from beef in older adults, thereby supporting the role of beans as a nutrient-dense source of protein and dietary fiber as part of a satisfying meal for older adults.

This trial was registered at clinicaltrials.gov as NCT05499819.

## Introduction

Overweight and obesity rates are of concern, with worldwide statistics indicating that 43% of the adult population were overweight and 16% were obese in 2022 [1]. Rates are higher in North America, with 2018–2019 statistics indicating that 60% of Canadian adults 18–79 y [2] and 74% of Americans ≥20 y were overweight or obese [3]. This prevalence is of concern since obesity is associated with an increased risk of many chronic diseases [1,[4], [5], [6], [7]], negatively impacts psychosocial factors, including quality of life and social stigma, and poses an economic burden from healthcare costs and reduced workplace productivity [6]. This evidence indicates a strong negative impact of overweight and obesity on health and society and emphasizes the need for feasible strategies to prevent weight gain and improve body weight management.

Obesity rates in older adults are also of concern, with North American statistics indicating that in 2018, 42.8% of adults ≥60 y were obese in the United States [8], and 28.1% of adults ≥65 y were obese in Canada [9]. These rates are of particular concern since the proportion of older adults is increasing [10], and increased age is a risk factor for many obesity-related chronic diseases and functional impairments [11,12]. Prevention of overweight and obesity is also warranted by evidence from a meta-analysis supporting an association between obesity and functional decline in older adults [13]. In addition to obesity risk, increased age presents physiological and social challenges to achieving adequate nutrition [14], as evidenced by Canadian data that identified a risk of nutrient deficiency in 34% of adults >65 y [15]. These considerations collectively warrant a focus on nutrient-dense approaches to prevent overweight and obesity in older adults.

Satiety enhancement is a proposed strategy to reduce risk of overweight and obesity [16] and is defined as the feelings of increased fullness and decreased hunger that prevent subsequent food intake after a meal [17]. Satiety can be measured using subjective evaluation of appetite sensations and objective measures of ad libitum food intake and 24-h energy intake [[17], [18], [19]]. Foods that promote satiety and decrease hunger may help individuals achieve energy balance through a delay of subsequent mealtimes [16,17], reduced energy intake at meals [16,20], smaller meal sizes, fewer temptations for opportunistic eating [16], and decreased impulsive eating behavior [21,22]. Satiety-promoting strategies may also improve adherence to healthy eating and body weight-management efforts by inducing intuitive feelings of fullness between meals [16,22]. This approach has advantages over restrictive weight loss dieting, which can present multiple challenges to weight loss [23] and has been associated with eating disorders [24] and poor diet quality [25]. Consequently, research that advances the understanding of the satiety potential of various foods, especially nutrient-dense foods, is an important contribution to effective body weight management to reduce risk of being overweight and obese.

Beans are a candidate food for satiety promotion due to their constituent protein and dietary fiber [26]. Protein has been widely studied for its role in satiety and body weight management, with evidence showing it may increase energy expenditure through diet-induced thermogenesis [[27], [28], [29], [30]], increase gluconeogenesis [[27], [28], [29], [30]], increase satiety hormones [[27], [28], [29], [30], [31]], alter amino acid concentrations and profile [[27], [28], [29], [30], [31]], and improve satiety when included in test meals [[30], [31], [32], [33]]. Dietary fiber has also been studied for its role in satiety and body weight management, with evidence showing it can add bulk to foods [[34], [35], [36]], promote gastric retention and distention [[34], [35], [36], [37], [38]], prolong digestive transit time to slow nutrient absorption [[34], [35], [36], [37], [38]], increase fecal energy loss [37], increase satiety hormones [35,37,38] and ferment to produce short-chain fatty acids [34,36].

Beans have been studied for their satiety potential, with evidence showing they are able to increase satiety [39,40], decrease prospective food intake [40], delay the return of hunger [41], and decrease the desire to consume more food [41]. Beans were also included in a meta-analysis of 9 studies that found a 31% increase in satiety from acute consumption of pulses [42]. Most of these studies included young, healthy adults, and the increasing prevalence of older adults [10] with increased obesity risk [12] justifies the need for satiety studies in older adults. In addition, since these studies mostly examined other pulses in comparison with simple carbohydrates, studies are needed that focus on beans and include comparators such as animal-based protein foods.

Beans can be compared with animal protein foods for satiety effects to support dietary guidance that promotes the intake of both plant and animal protein foods [19,43]. Studies that have compared plant protein foods to animal protein foods support their ability to improve satiety to a similar [[44], [45], [46]] or greater [47] extent than animal protein foods, although studies are limited, and only 1 study has focused on beans [44]. This narrow scope of literature warrants more satiety studies that focus on beans, especially in older adults who are a growing segment of the population [10] and have an elevated risk of chronic diseases and functional impairments that are exacerbated by obesity [11,12]. Therefore, the current study was designed to compare the effects of black beans, red kidney beans, and extra-lean ground beef on satiety-related measures in older adults.

## Methods

### Study design and approvals

This study (NCT05499819) used a randomized, crossover design in which participants completed 3 study visits separated by ≥1-wk washout periods. Data collection occurred at the Human Nutraceutical Research Unit (HNRU) at the University of Guelph, and the study was reviewed and approved by the University of Guelph research ethics board (REB 22-03-028).

### Participant recruitment and screening

Participants were recruited from older adult communities in Guelph, Ontario, Canada, and surrounding areas using posters, newspapers and website advertisements, and social media. Participants were included if they were males or females ≥60 y old, community-dwelling, and had a BMI between 18.5 and 30 kg/m^2^. Exclusion criteria included a diagnosed digestive-related condition (i.e., Celiac disease, constipation, diverticulitis, gastritis, gastroesophageal reflux disease, gluten intolerance, hemorrhoids, inflammatory bowel disease, irritable bowel syndrome, lactose intolerance), a diagnosed cognitive-related condition (i.e., Alzheimer’s disease, amnesia, dementia, generalized anxiety disorder, major depressive disorder, Parkinson’s disease, schizophrenia, traumatic brain injury), any other medical condition that did not have stable (≥3 mo) management, unstable (<3 mo) use of medications or natural health products, a medical or surgical event requiring hospitalization (within 3 mo), not vaccinated for COVID-19 (≥2 doses), use of tobacco, cannabis, or vape, recent (within 3 mo) or intended significant (>4 kg) weight loss or gain, frequent breakfast skipping (≥4 d/wk), pulse consumption >4 servings/wk (1 serving = ¾ cup or 135 g), vegan diet, alcohol consumption >14 drinks/wk or >4 drinks/sitting (1 drink = 14 g ethanol), anaphylactic food allergy, dislike of study foods (beans, ground beef, shredded cheddar cheese, white tortillas, frozen cheese pizza), three-factor eating questionnaire scale scores >11 for cognitive restraint, >9 for disinhibition, or >8 for hunger [48], and overnight shift workers.

Participants were screened using an initial brief telephone questionnaire (screening-1) followed by an in-person screening visit (screening-2) at the HNRU. Screening-2 involved measurement of body weight and height, completion of a detailed eligibility questionnaire, completion of the three-factor eating questionnaire, and a presentation that reviewed the study details. Eligible and interested participants completed a study orientation where they were provided with a study handbook, had all their questions answered, and provided written consent before starting the study. A sample size of 35 participants was determined in accordance with the satiety methodology guidance literature [49].

### Study treatments and test meals

Study treatments included canned black beans (beans-BL), canned red kidney beans (beans-RK), and extra-lean ground beef that were incorporated into a breakfast tortilla bake test meal (Table 1). The beans-BL and beans-RK treatments were provided in the amount of ¾ cup (135 g) to reflect Health Canada’s most recent ¾ cup serving size for beans [50] and to be within the ½–1 cup servings of beans examined in the previous pulse satiety studies [42]. The beef treatment was provided in the cooked amount of 80 g (⅔ cup) to reflect Health Canada’s most recent 75 g serving size for cooked, lean meat [50] and to be comparable to the estimated amounts of 80–85 g beef examined in previous satiety studies [44,45]. To comply with satiety research best practices, the test meals were designed to be appropriate for a breakfast meal [17] and were weight-matched using variable amounts of water (345–400 mL) served with the test meals [17,51]. The inherent differences in the nutritional composition of the beans and beef resulted in the beans-BL and beans-RK test meals providing less protein (21.4 g) and more dietary fiber (11.9 g) than the beef test meal (34.9 g and 1.7 g, respectively) (Table 1). The order of the study treatments was randomized using an online randomizer tool (https://www.randomizer.org), and neither researchers nor participants were blinded to the whole-food treatments.

**TABLE 1:** Recipe ingredients and nutritional composition for the black bean, red kidney bean, and extra-lean ground beef test meals.

	Beans-BL	Beans-RK	Beef
Ingredients^1^			
Beans-BL, drained and rinsed (g)	135		
Beans-RK, drained and rinsed (g)		135	
Extra-lean ground beef, pan-fried (g)			80
Dempster’s original large (10”) tortilla [*n* (g)]	1 (61)	1 (61)	1 (61)
President's Choice triple cheddar shredded cheese blend (g)	20	20	20
President's Choice mild salsa (g)	40	40	40
Water (g)	345	345	400
Nutritional composition^2^			
Weight (g)	601	601	601
Energy (kcal)	426	426	440
Protein (g)	21.4	21.4	34.9
Fat (g)	12.6	12.6	19.4
Carbohydrates (g)	59.0	59.0	30.6
Dietary fiber (g)	11.9	11.9	1.7

Abbreviations: Beans-BL, canned black beans; Beans-RK, canned red kidney beans.

1 Study treatments and test meal ingredients were sourced from the local grocer.

2 Nutrient information was obtained from the Canadian Nutrient File for all study treatments and test meal ingredients except the tortilla, which was obtained from the product packaging due to lack of data.

Breakfast tortilla bake test meals were prepared the morning of the study visit. Bean treatments were mashed with a fork, and the beef was pan-fried and then placed on 1 side of the tortilla with shredded cheddar cheese on top. The tortilla was folded in half, baked at 375°F for 10 min, and served with optional mild salsa for dipping. Participants were served the test meal and water in a private sensory booth with instructions to consume within 15 min.

### Data collection and analysis

All study visits occurred at the HNRU, where participants arrived in the morning following an overnight (10–12 h) fast with no food or water; however, tooth brushing and consuming water with medications, if needed, were permitted. Participants were instructed to consume a consistent dinner meal the evening prior and to maintain their habitual lifestyle, including dietary and physical activity habits throughout the entire study. For 24 h before each study visit, they were instructed to avoid the consumption of pulses, beef, and alcohol, as well as over-the-counter medications and unusual physical activity. Fasting body weight was measured in duplicate to the nearest 0.1 kg using an electronic scale (Acculab Sartorius Group SVI-200F) after participants had removed their shoes and pocket contents.

Satiety was measured using a 1-page paper questionnaire that asked participants to rate their subjective appetite sensations of fullness, satisfaction, hunger, desire to eat, and prospective food consumption using a 100 mm visual analog scale (VAS), a validated and reproducible measure of subjective satiety assessment [49]. Participants were provided with a ruler and instructed to clearly mark a straight vertical line along the horizontal VAS. Satiety questionnaires were completed at baseline and 15 min after the start of the breakfast test meal consumption in a private sensory booth and then given again at 30, 45, 60, 90, 120, 150, and 180 min after the breakfast test meal consumption in a quiet room. During this time, participants could read, listen to music, or browse the internet but were asked to refrain from speaking or reading about food or the study proceedings on their own or with others.

Satiety questionnaire VAS responses were measured to the nearest 0.1 mm using a ruler. Responses over all time points were summarized with the calculation of total AUC, the primary outcome measure, using GraphPad Prism (GraphPad Software Inc). Overall appetite score AUC was calculated by adapting the formula by Gibbons et al. [52] to include satisfaction over a total of 5 scales: fullness + satisfaction + (100 – hunger) + (100 – desire to eat) + (100 – prospective food consumption) / 5.

Palatability of the breakfast test meals was assessed immediately after consumption using a VAS that participants completed to rate their liking of taste, texture, and overall pleasantness. Palatability sensation VAS responses were measured to the nearest 0.1 mm using a ruler.

Food intake was measured at an ad libitum pizza and water lunch meal provided 180 min after the breakfast test meal. Participants sat in private sensory booths and were served a plate containing 220 g of irregularly sliced frozen Delissio thin crust 4-cheese pizza (Nestlé) that was prepared in the HNRU metabolic kitchen oven according to the manufacturer’s instructions. Participants were instructed to consume as much pizza as they liked from the plate over a 6-min period, after which a fresh plate of 220 g pizza was provided until the participant passed a “Comfortably full” card to the researcher. Plates were weighed to the nearest 0.01 g using an electronic scale (Denver instrument S-2002) before and after consumption to calculate the total amount of food consumed, which was converted to total energy intake using the nutrition information from the pizza package.

Water was also provided to participants in the amount of 500 g in a glass to drink with their pizza. Participants were instructed that they could request more water by passing a “More water please” card to the researcher. Total water intake was calculated by weighing the glass before and after consumption using an electronic scale (Denver instrument S-2002).

Energy intake for the rest of the day was assessed using a weighed food record. Participants were sent home with an electronic food scale (Starfrit Electronic Kitchen Scale) and were instructed to record the description and weight or measure of everything they ate or drank from the time they left the HNRU to the end of their day. Completed food records were reviewed with participants for clarity and inputted into the ESHA food processor nutrition analysis software program (version 11.5.226, database 11.5.0) for analysis of energy intake. Twenty-four-hour energy intake was then determined by summing the energy intake from the breakfast test meal, the lunch pizza meal, and the weighed food record.

### Data and statistical analysis

Data were entered into Microsoft Excel worksheets and checked for normality using box plots and stem leaf diagrams. Statistical analyses were completed using the Statistical Analysis System (version 9.4, SAS Institute), with *P* < 0.05 considered significant. Participant body weight, overall appetite score, treatment palatability sensation scores, food and water intake, and 24-h energy intake were compared between treatments using repeated-measures analysis of variance, followed by a Tukey’s test for multiple comparisons. Appetite sensation total AUCs were compared between treatments using repeated-measures analysis of covariance, including baseline as a covariate, followed by a Tukey’s test for multiple comparisons. Data are reported as mean ± SE unless otherwise indicated.

## Results

### Participants

**FIGURE 1:**
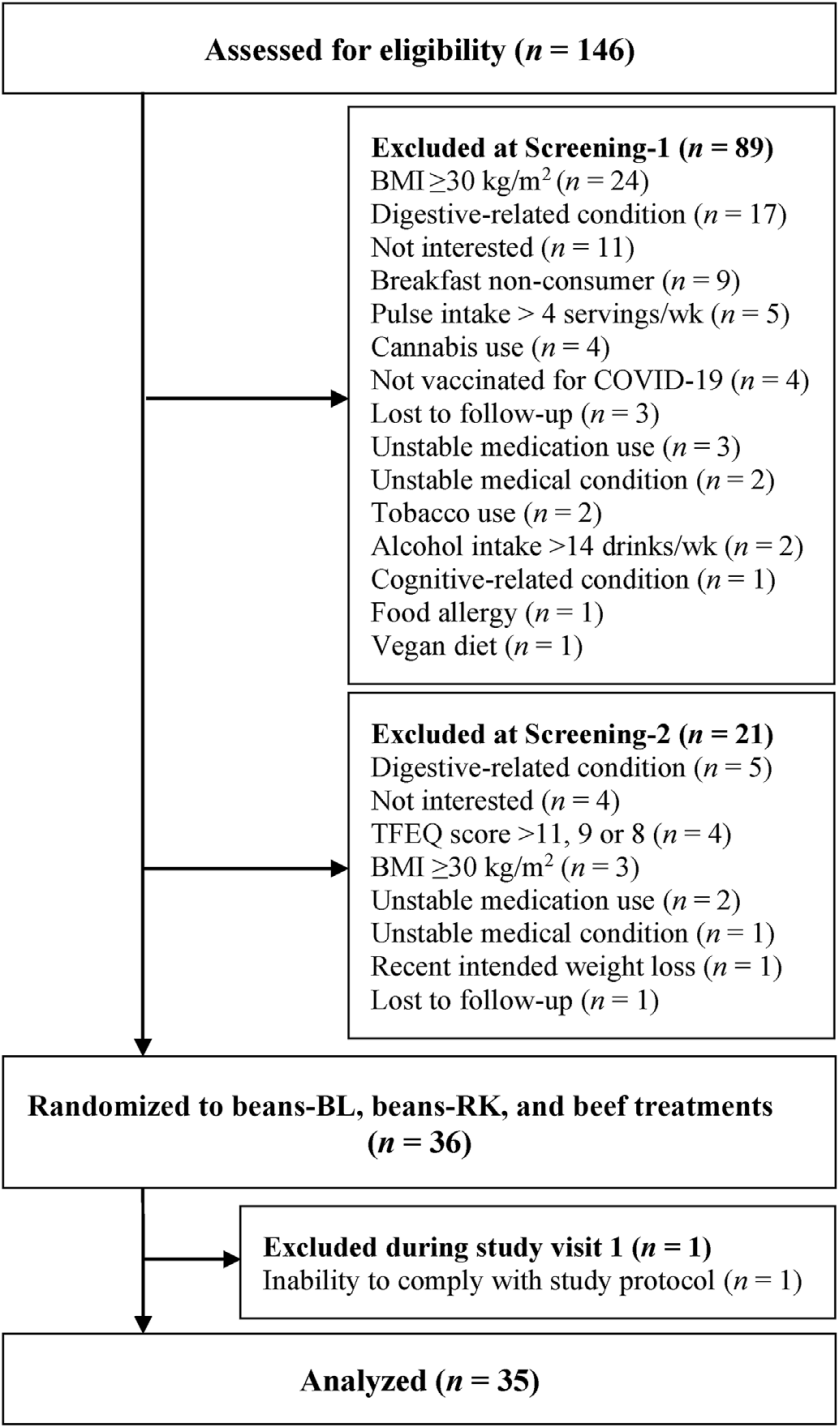
CONSORT diagram summarizing participant flow through the study. Beans-BL, canned black beans; beans-RK, canned red kidney beans; BMI, body mass index; CONSORT, consolidated standards of reporting trial; COVID-19, coronavirus disease 2019; TFEQ, three-factor eating questionnaire.

Participant flow included 146 individuals who were screened, of which 36 were eligible and interested (Figure 1). One participant was excluded during study visit 1 due to an inability to comply with the study protocol, which resulted in a total of 35 participants who completed the study and were included in the analysis (Figure 1).

Participant baseline characteristics are summarized in Table 2. During the study, body weight and BMI did not significantly differ between the beans-BL, beans-RK, or beef treatments (data not shown).

**TABLE 2:** Participant baseline characteristics^1^.

Characteristic	Value
Sex	
Female	20 (57.1)
Male	15 (42.9)
Age (y)	72.4 ± 6.66
Body weight (kg)	70.7 ± 13.0
Height (cm)	167.5 ± 9.70
BMI (kg/m^2^)	25.1 ± 3.25
Ethnicity	
White/European	33 (94.3)
Other	2 (5.7)
Number of health conditions	1.2 ± 1.07
Number of medications	1.8 ± 1.85
Number of NHPs	2.4 ± 1.56
TFEQ scores	
Cognitive restraint	6.3 ± 2.87
Hunger	3.3 ± 1.81
Disinhibition	3.1 ± 1.92

Abbreviations: BMI, body mass index; NHP, natural health product; SD, standard deviation; TFEQ, three-factor eating questionnaire.

1 Values are mean ± SD or *n* (%), *n* = 35.

### Subjective appetite sensations

**FIGURE 2:**
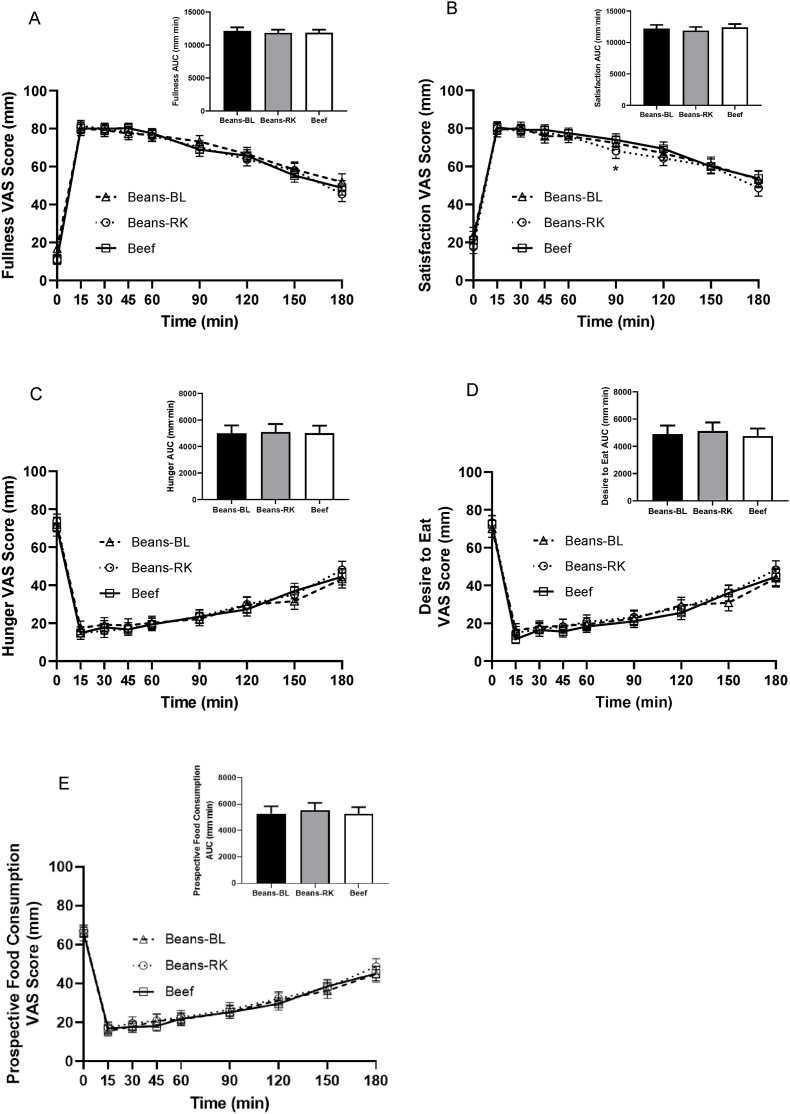
VAS scores over 180 min and total AUCs for fullness (A), satisfaction (B), hunger (C), desire to eat (D), and prospective food consumption (E) after older adults (*n* = 35) consumed beans-BL, beans-RK or beef treatments. Data are mean ± SE. Significant differences between timepoints within treatments are not shown for clarity. The beef was provided as extra-lean ground beef. AUC, area under the curve; beans-BL, canned black beans; beans-RK, canned red kidney beans; SE, standard error; VAS, visual analog scale.

Subjective appetite sensation mean scores significantly changed over time within the beans-BL, beans-RK, and beef treatments (Figure 2). Fullness and satisfaction mean scores significantly increased following consumption of the test meal (0–15 min), remained increased until 90 min or 120 min, and then gradually decreased to 180 min, at which point they were still significantly higher than baseline for all treatments (Figure 2A and B). Hunger, desire to eat, and prospective food consumption mean scores significantly decreased following consumption of the test meal (0–15 min), remained decreased until 90 min or 120 min, and then gradually increased until 180 min, at which point they were still significantly lower than baseline for all treatments (Figure 2C, D, and E). Subjective appetite sensation mean scores at individual timepoints were not significantly different between treatments (Figure 2).

Subjective appetite sensation score total AUCs for beans-BL, beans-RK, and beef were not significantly different between treatments for fullness (*P* = 0.84) (Figure 2A), satisfaction (*P* = 0.33) (Figure 2B), hunger (*P* = 0.98) (Figure 2C), desire to eat (*P* = 0.53) (Figure 2D), or prospective food consumption (*P* = 0.61) (Figure 2E). Overall appetite score was also not significantly different between treatments (*P* = 0.58) (data not shown).

### Food and water intake at the ad libitum lunch meal and 24 h energy intake

Pizza and water intake at the 180 min ad libitum lunch meal were not significantly different between beans-BL, beans-RK, and beef (*P* = 0.07 and *P* = 0.63, respectively) (Table 3). Twenty-four-hour energy intake was also not significantly different between treatments for beans-BL, beans-RK, and beef (*P* = 0.90) (Table 3).

**TABLE 3:** Food and water intake at the ad libitum pizza lunch meal and 24-h energy intake for older adults consuming black bean, red kidney bean, and extra-lean ground beef test meals^1^.

	Beans-BL	Beans-RK	Beef
Pizza intake (g)	167.5 ± 14.2	175.8 ± 13.9	159.5 ± 13.0
Pizza intake (kcal)	366.5 ± 31.0	384.5 ± 30.4	349.0 ± 28.4
Water intake (mL)	236.0 ± 29.7	255.0 ± 27.6	235.6 ± 26.7
24-h energy intake (kcal)	1877 ± 86.9	1861 ± 75.5	1890 ± 80.3

Abbreviations: Beans-BL, canned black beans; Beans-RK, canned red kidney beans; SE, standard error.

1 Values are mean ± SE, *n* = 35.

### Treatment palatability

Taste, texture, and pleasantness mean scores were >60.0 mm (on a 100 mm VAS scale) for beans-BL, beans-RK, and beef (Table 4). Taste and pleasantness were significantly lower for beans-BL compared to beef, while texture was not significantly different between any of the treatments (Table 4).

**TABLE 4:** Palatability sensation scores for older adults consuming black bean, red kidney bean, and extra-lean ground beef test meals^1^.

	Beans-BL	Beans-RK	Beef
Taste (mm)	63.7 ± 4.74^a^	68.2 ± 3.37^ab^	74.3 ± 3.25^b^
Texture (mm)	65.3 ± 4.67	66.1 ± 3.97	69.2 ± 4.01
Pleasantness (mm)	60.6 ± 4.64^a^	67.0 ± 4.04^ab^	71.6 ± 3.65^b^

Abbreviations: Beans-BL, canned black beans; Beans-RK, canned red kidney beans; SE, standard error.

1 Values are mean ± SE, *n* = 35. Values with different superscripts within a row are significantly different from those of treatments that are compared using repeated-measures ANOVA (analysis of variance) followed by a Tukey’s test for multiple comparisons.

## Discussion

This study compared the effects of 2 canned bean varieties and beef on satiety-related measures in a sample of older adults to help identify strategies that could contribute to overweight and obesity prevention and management in this under-studied demographic. Participants consumed each of the 3 treatments in a breakfast test meal following a randomized, crossover study design that included outcome measures of subjective appetite sensations, food intake from an ad libitum pizza lunch, and 24-h energy intake. This research expands a limited literature of satiety effect comparisons between plant and animal protein foods [[44], [45], [46], [47]] by including older adults and comparing 2 varieties of beans (with their protein and dietary fiber content) and beef (with its protein content). Study test meals were designed to reflect normative serving sizes of these whole foods to facilitate the application of the results to realistic mealtime situations, for example, when a serving of beef is replaced with a serving of beans as a plant-based meat alternative.

The focus on older adults in the current study broadens the scope of studied participant groups in the beans and satiety literature, which is important since older adults are a fast-growing proportion of the population [10] and are at an elevated risk for obesity-related chronic diseases and functional impairments [11,12]. Although most studies in the bean and satiety literature have been conducted in younger adults of a broader age range without health conditions [44,46,47], participants in the current study were ≥60 y old (72.4 ± 6.66), had a mean of 1.2 health conditions and used a mean of 1.8 medications (Table 2). The need for studies in participants at different life stages, such as older adulthood, is reinforced by a recent workshop by the National Academy of Sciences, Engineering, and Medicine that examined how nutrition changes over a person’s life course [53] and a recent National Academy of Sciences, Engineering, and Medicine publication on how the use of the dietary reference intakes for healthy individuals may be excluding many who live with chronic disease [54]. Overall, the current study addresses the need for research in a diversity of life stages and health statuses, thereby increasing the applicability of the bean and satiety literature to the older adult population.

Results of the current study showed that fullness and satisfaction significantly increased while hunger, desire to eat, and prospective food consumption significantly decreased after consumption of all breakfast test meals, including the black beans, the red kidney beans, and the beef. It is noteworthy that the beans and beef treatments did not significantly differ for these appetite responses, nor did they differ for food intake at the ad libitum lunch meal or 24-h energy intake, despite the bean test meals providing relatively less protein (21.4 g) and more dietary fiber (11.9 g) and the beef test meal providing relatively more protein (34.9 g) and less dietary fiber (1.7 g). This evidence suggests that the higher dietary fiber present in the bean treatments contributed to their satiety-promoting effects and compensated for their lower-protein content relative to the beef treatment.

Protein and dietary fiber have both been studied for their mechanistic contributions to satiety. Protein digestion has a high metabolic cost that results in dietary-induced energy expenditure, which may increase satiety through higher body temperature and increased oxygen demand for protein metabolism [27,30]. Protein intake has also been shown to increase satiety hormones and amino acids that can influence gut-brain feedback mechanisms related to satiety [29,30]. At the same time, dietary fiber can slow oral processing and digestive transit time, prolong nutrient absorption, increase gastric retention and distention, and stimulate the release of satiety hormones and short-chain fatty acids [36,37]. These combined satiety-related mechanisms of protein and dietary fiber may be complementary in their effects on satiety following the consumption of foods that contain varying amounts of both nutrients. This idea is relevant and generalizable since a realistic whole-food diet includes foods that contain both protein and dietary fiber, which reinforces the need to consider their combined influence on satiety.

Appetite sensation AUCs did not significantly differ between black beans, red kidney beans, and beef in the current study, which is generally consistent with previous studies that have compared beans to animal foods on satiety-related measures. Most related is a study that also compared beans (17 g protein, 12 g dietary fiber) to beef (26 g protein, 3 g dietary fiber) in 28 adults of a broader age range (18–65 y old) and found no significant differences in appetite sensation AUCs [44]. Another study that compared meals containing legumes (fava beans and split peas; 18 g protein, 10 g dietary fiber) or meat (veal and pork; 39 g protein and 6 g dietary fiber) in 43 adults, 18–40 y old, found no significant differences in AUCs for fullness, satisfaction, hunger, prospective food consumption, or desire to eat something sweet, salty, fatty, or meaty/fishy [47]. However, the same study also included a third high-protein legume meal that was comparable in protein content (38 g) to the meat meal and higher in dietary fiber content (25 g) than the lower-protein legume meal and found that it caused significantly greater fullness, lower hunger, lower prospective food intake, and lower overall appetite compared to the lower-protein legume and the meat meals. By combining higher amounts of dietary fiber (similar to legumes) and protein (similar to meat) into 1 treatment, the significant satiety effects of this high-protein legume meal support the additive influences that dietary fiber and protein can have on satiety response. Nielsen et al. [46] also matched a plant (fava beans and split peas) meal and a meat (veal and pork) meal for protein and dietary fiber content by adding pea fiber to the meat meal and found that appetite sensation AUCs and overall appetite score were not different between the 2 meals in 33 adult males, 18–50 y old. Another study conducted in 21 adults, 18–30 y old, matched soy protein and beef pasta meals for energy, protein, and dietary fiber content (34 g protein, 2 g dietary fiber) and found no difference in fullness or hunger AUCs [45]. The same study also matched soy protein and beef sandwich meals for serving size (24 g and 34 g protein and 5 g and 1 g dietary fiber, respectively), which is most comparable to the current study, and again did not find any differences in fullness or hunger [45]. Overall, these results reinforce the relative contribution of protein and dietary fiber to satiety and the idea that the higher dietary fiber content of beans relative to meat and the higher protein content of meat relative to beans results in a comparable subjective satiety response as measured by appetite sensations.

Subjective appetite sensations are the typical assessment used to determine feelings of satiety [18], but they can be supplemented by objective measurement of food intake at a subsequent meal and energy intake for the remainder of the day [18,20]. The current study’s results of no significant differences between the beans and the beef for food intake at the ad libitum pizza lunch meal and energy intake for the remainder of the day reflect findings of previous studies that have compared plant (beans or soy) and meat treatments. Bonnema et al. [44] showed no significant differences between bean and beef meatloaf test meals for ad libitum food intake at a subsequent meal of vending machine snacks and energy intake for the remainder of the day. Kristensen et al. [47] also found no significant difference in ad libitum food intake at a subsequent pasta Bolognese meal between legume (fava beans and split peas) and meat (veal and pork) patties; however, when the legume meal was supplemented with protein, food intake was significantly lower than both the lower-protein legume and meat meals, corresponding with the appetite sensation results [47]. Similarly, Nielsen et al. [46] reported no significant differences in ad libitum pasta Bolognese intake or energy intake for the remainder of the day between plant (fava beans and split peas) and meat (veal and pork) meals that were matched for protein and dietary fiber content. Finally, Douglas et al. [45] found no significant differences between soy and beef pasta meals matched for energy, protein, and dietary fiber content or for sandwiches matched for serving size, in the time to request their next meal, for food consumed at the ad libitum dinner buffet (crackers, fruits, vegetables, lunch meats, and cheese), or for energy intake for the remainder of the day. Overall, these food and energy intake results are consistent with the subjective appetite sensation results of the same studies and with the results of the current study. These data collectively contribute evidence that beans are as capable as beef in managing food and energy intake throughout the day and support the addition of beans to meals to help control daily energy intake through satiety-mediated effects.

The palatability of foods is also important to consider since results cannot be translated into practice if the foods are not accepted. Palatability results of the current study showed that black beans (but not red kidney beans) were less well-received than beef for taste and pleasantness, whereas texture was not significantly different between any of the treatments. Previous studies have seen inconsistent palatability results, with some finding legume-based test meals were less palatable [46,47] or not different in palatability [44,45,47] compared to meat-based treatments, although these studies have also varied in their meal ingredients. Palatability is relevant since highly palatable foods can influence eating behavior, such as increasing self-reported appetite and earlier recovery of appetite after a meal [55]. Palatability also influences the release of dopamine and serotonin to increase the hypothalamic reward system [56]. Evidence from this study suggests that efforts are needed to facilitate older adults’ acceptance of beans so they can consider them an option for a nutrient-dense and satisfying meal.

Limitations of the current study include its generalizability due to the low ethnic diversity of the participants and the lack of blinding to the whole-food study treatments; however, the data were coded to enable blinding during the data entry and analysis. Another limitation is the use of a self-reported food record, which could limit accuracy; however, participants were provided with detailed instructions along with an electronic food scale to improve accuracy. The strengths of this study include its randomized, crossover design, which included a thorough screening process and data collection that adhered to satiety methodology best practices [17]. Another strength is the focus on older adults, who represent an under-studied segment of the population despite their proportionally higher representation in the population [10]. Finally, the whole-food nature of the treatments that were provided in a serving size amount increases the applicability of the results, and the matching of the test meals for weight enables the results to be included as evidence for consideration of a satiety health claim [17,51].

In conclusion, black beans, red kidney beans, and beef increase fullness and satisfaction while reducing hunger, desire to eat, and prospective food consumption with no significant differences between them. These results support the idea that the higher dietary fiber content in beans compensated for their lower-protein content compared to beef, suggesting that a combination of dietary fiber and protein can additively contribute to the satiety response. These findings highlight the potential for beans to act as a plant-based alternative or complement to beef for managing satiety and energy intake, particularly for older adults at risk of obesity-related chronic diseases. Implications of this research should consider the combination of beans and beef in a single meal or food product. It is relevant to consider that plant-based meat alternatives that imitate meat products have increased in popularity [57,58]. Results of the current study provide a rationale for considering products that combine plant-based protein with meat, such as a burger patty made from both black beans and meat. In developed countries, including North America, Australia, and many European countries, meals are often centered around large portions of meat, making them some of the top carnivorous countries in the world [59,60], with the United States and the United Kingdom consuming almost 3 times and double the global average of meat per capita, respectively [60]. Including more plant-based protein foods in meals while reducing but not eliminating meat can facilitate a transition to a more plant-based diet while still appreciating cultural and traditional values related to meat. The combination of beans and beef has the advantage of a diverse and complementary nutrient profile that includes both high dietary fiber and high protein. Therefore, this research can advocate for the amalgamation of plant-based proteins with meat products to create a nutritionally balanced and satisfying meal for older adults. Overall, continued efforts are warranted to facilitate acceptance of beans by older adults so that beans can be considered as a whole-food source of protein and dietary fiber and as an option to be included in a nutrient-dense and satisfying meal.

## Author contributions

The authors’ responsibilities were as follows–MJF, BFA, AMD: designed the research; MJF, BFA, ZJR, AMD: conducted the research; MJF, BFA, ZJR, AMD: analyzed the data; MJF, BFA, AMD: wrote the paper; AMD: had primary responsibility for final content; and all authors: read and approved the final manuscript.

## Data availability

Not applicable.

## Funding

This work was supported by the Ontario Bean Growers.

## Conflict of interest

AMD is an editorial board member for The Journal of Nutrition and played no role in the journal’s evaluation of the manuscript. All other authors report no conflicts of interest.
